# Supplementary material for: Extending density functional theory with near chemical accuracy beyond pure water
Source: Nat Commun. 2023 Feb 13;14:799. doi: 10.1038/s41467-023-36094-y (PMC9925738; doi:10.1038/s41467-023-36094-y)
Supplement: Supplementary file 3 — Supplementary Information [file 41467_2023_36094_MOESM3_ESM.pdf]

Supplementary Information for Extending density functional theory with near chemical accuracy beyond pure water

Suhwan Song[a,b], Stefan Vuckovic[c,d], Youngsam Kim[a], Hayoung Yu[a], Eunji Sim[a,*], and Kieron Burke[b,e]

[a]Department of Chemistry, Yonsei University, 50 Yonsei-ro Seodaemun-gu, Seoul 03722, Korea

[b]Department of Chemistry, University of California, Irvine, CA 92697, USA

[c]Institute for Microelectronics and Microsystems (CNR-IMM), Via Monteroni,Campus Unisalento, 73100 Lecce, Italy

[d]Departments of Chemistry&Pharmaceutical Sciences and Amsterdam Institute of Molecular and Life Sciences (AIMMS), Faculty of Science, Vrije Universiteit, De Boelelaan 1083, 1081HV Amsterdam, The Netherlands

[e]Departments of Physics&Astronomy, University of California, Irvine, CA 92697, USA

January 4, 2023

Supplementary Note 1. DFT and DC-DFT

In KS-DFT [1], the ground state energy is obtained by minimizing the following functional over densities,

$$E_v[n] = T_{\rm S}[n] + U_{\rm H}[n] + E_{\rm XC}[n] + \int d^3r\, n(\mathbf{r})v(\mathbf{r}), \qquad (1)$$

where the minimizing $n_v(\mathbf{r})$ is the ground-state density, $T_{\rm S}[n]$ is the KS noninteracting kinetic energy functional, $U_{\rm H}[n]$ the Hartree energy, and $E_{\rm XC}[n]$ is the exchange-correlation (XC) functional. In practical KS calculations, the only term that of Eq. 1 that is approximated is $E_{\rm XC}[n]$. Whatever approximation we use for XC, it yields an error in both energies and the densities, as the minimizing density in Eq. 1 is different from the exact when an approximate XC functional is used in place of its exact counterpart. We can write the error of any KS calculation as: $\Delta E = \tilde{E}[\tilde{n}] - E[n]$ as, where $E[n]$ is the exact functional (Eq. 1) and n is the exact density (we drop the v subscript for brevity), while tildes denote approximate quantities. The main idea of DC-DFT is to separate the errors in ΔE into a functional error, which is present even with the exact density [2]:

$$\Delta E_{\rm F} = \tilde{E}[n] - E[n] = \tilde{E}_{\rm XC}[n] - E_{\rm XC}[n], \qquad (2)$$

and the density-driven error is the remainder of ΔE:

$$\Delta E_{\rm D} = \tilde{E}[\tilde{n}] - \tilde{E}[n]. \qquad (3)$$

In most KS-DFT calculations, $\Delta E_{\rm F}$ strongly dominates ΔE, implying that approximate KS densities are very good as measured by the impact on energies. A very simply example for such case is the total energy of the helium atom, whose energy computed from the PBE functional[3] barely changes after the PBE density is replaced with the exact one.[2] However, for a significant number of chemical domains (e.g., anions and barrier heights), $\Delta E_{\rm D}$ can be much larger than $\Delta E_{\rm F}$[4, 5]. A good example for such a case is H^- with the PBE functional, which gives excellent energies when evaluated on the exact densities, whereas the self-consistent PBE density cannot even bind two electrons for this anion.

*esim@yonsei.ac.kr

Supplementary Note 2. Spotting and curing large density-driven errors

The following two questions are of key importance in DC-DFT: (i) How do we spot cases where $\Delta E_{\rm D}$ is large?; (ii) What do we do in such cases to reduce large density-driven errors? With access to exact densities we can easily answer (i), as with those we can easily measure $\Delta E_{\rm D}$ by Eq. 3. At the same time, we answered (ii), as $\Delta E_{\rm D}$ entirely vanishes with exact densities. However, exact densities are available only for small systems [2, 6], and if we always had access to exact energies and densities from highly accurate wavefunction theories, we would not even bother with KS-DFT. Thus one needs to find more practical ways to answer (i) and (ii). In relation to (i), the following quantity has been introduced:

$$\tilde{S} = \left|\tilde{E}[n^{\rm LDA}] - \tilde{E}[n^{\rm HF}]\right|, \qquad (4)$$

and is called *density sensitivity*. $\tilde{S}$ requires two nonempirical densities: the HF densities which are typically overlocalized and the local density approximation (LDA) densities which are typically delocalized. $\tilde{S}$ serves as a practical measure of density sensitivity of a given reaction and approximate functional. For small molecules, $\tilde{S}$ greater than the heuristic cutoff of 2 kcal/mol implies density sensitivity, indicating that the calculation may suffer from a large $\Delta E_{\rm D}$. What should we do then to reduce large $\Delta E_{\rm D}$? In these cases, evaluating an approximate functional on the HF in place of self-consistent densities will likely reduce $\Delta E_{\rm D}$ and likely improve the functional's performance. This procedure, called HF-DFT, is the practice of evaluating an XC approximation on the HF density and orbitals. It had been used a long before DC-DFT was proposed [7, 8, 9, 10, 11] but only because HF densities were more convenient than self-consistent densities. KS-DFT does not always benefit from HF densities (e.g, cases where $\Delta E_{\rm D}$ is small) and in Refs. [12, 13, 14] we discuss in more details formal and practical (dis)advantages of HF-DFT over SC-DFT.

Supplementary Note 3. HF-DFT and related DC-DFT procedures

Following the idea that in some cases HF-DFT works better and SC-DFT in others, DC(HF)-DFT has been proposed [13, 14]. It is a procedure that discriminately uses HF densities, as DC(HF)-DFT becomes HF-DFT for cases that are both density-sensitive ($\tilde{S}$ above a given cut-off value, 2 kcal/mol as discussed in Ref. [6].) and whose HF solution is not severely spin-contaminated. Otherwise, DC(HF)-DFT reverts to SC-DFT. Since HF-DFT uses the HF density as a proxy for the exact density, we only use it when there is little or no spin contamination. We calculate the expectation values of the spin-squared operator, S^2, and only use the HF density if the $<S^2>$ from the HF calculation deviates less than 10% from the exact $<S^2>$ as discussed in Refs. [13] and [15]. Otherwise, we use the self-consistent density. As it combines the best of both of them, DC(HF)-DFT comes with a range of advantages over both HF-DFT and SC-DFT as further detailed in Ref. [13]. These advantages come at a small extra cost, as for DC(HF)-DFT we need to run up to three distinct self-consistent cycles to obtain the three densities (the SC density for a given functional and those from HF and LDA needed to calculate $\tilde{S}$). While we consider DC(HF)-DFT the state-of-the-art DC-DFT-based procedure, in the present work we want a simple DC framework that can be applied easily and routinely. r^{2}SCAN and SCAN are general-purpose functionals and the ease of their use would be undermined if tandem with DC(HF)-DFT, which would require always calculating $\tilde{S}$ and possibly making adjustments to its cut-off value. For this reason and encouraged by the very good performance of HF-DFT with SCAN-like functionals[16, 17], we employ HF-DFT as a DC-DFT procedure throughout this work. As said, constructing the robust and accurate HF-r^{2}SCAN-DC4 is the central objective of this work. While the resulting HF-r^{2}SCAN-DC4 can be routinely used by applying it to HF orbitals without ever needing to calculate $\tilde{S}$ of a given reaction, the use of $\tilde{S}$ is vital for our training of HF-r^{2}SCAN-DC4. Specifically, we use density-sensitivities of the training reactions to fit the D4 part of HF-r^{2}SCAN. Further technical details of this fitting procedure will be given in the next section.

Supplementary Note 4. Optimizing dispersion parameters

D4 stands for the generally applicable atomic-charge dependent London dispersion correction term developed by Grimme and co-workers.[18]. It has 4 functional-dependent parameters s_6, s_8, a_1, and a_2. Following Refs. [19] and [18], we set s_6 to unity as is common for functionals that do not capture long-range dispersion interactions. We optimized the s_8, a_1, and a_2 parameters by minimizing the mean absolute error (MAE) for the density-insensitive GMTKN55 reactions by following the DC-DFT ideas of Ref. [12]. However, the density-insensitive reactions in GMTKN55 largely fall into two distinct parameter groups for HF- r^{2}SCAN: s_8 has a negative value for noncovalent interactions, but is positive for the rest. The difference in MAE of density-insensitive cases between those two groups is miniscule (below 0.01 kcal/mol). For example, (s_8,a_1,a_2)=(-0.20,0.07,6.50) gives 1.209 kcal/mol for the density-insensitive MAE while (0.39,0.09,7.02) gives 1.210 kcal/mol. Such a difference is not meaningful. Small changes in computational details such as DFT grid information, two-electron operator fitting scheme, etc. changes the values of the parameters, since reaction energy errors and density sensitivity values can be changed by 0.01 kcal/mol with those changes. To eliminate this ambiguity while ensuring accuracy in water interactions, we include the density-insensitive water$\cdots$water pair interaction energy as a validation set. The two most stable water hexamers, the prism and the cage, are used to calculate the water$\cdots$water 2-body interaction energy error per dimer, relative to CCSD(T)/CBS in Ref. [20]. We multiply its weight by 7 in our loss function to produce a better defined minimum and regularize the result (if we used 1, it has no effect; if we used 1000, we simply fit to this data). We can rationalize this value by noting that the mean density sensitivity of these pairs is 0.27 kcal/mol, which is about 1/7th of our density sensitivity cutoff. The resulting values for the three parameters are: -0.36, 0.23, 5.23 for s_8, a_1, and a_2 each.

Supplementary Note 5. Additional results for the GMTKN55 database

In Table 1, we list MAE (kcal/mol) of SC-r^{2}SCAN and HF-r^{2}SCAN functional with and without the dispersion correction for the chemically diverse GMTKN55 database [21]. def2-QZVPPD basis set is used.

Supplementary Note 6. Additional results for water clusters

i. MD generated dimer structures

The structures used in Figures 1(e) and 2(b), have been obtained from the molecular dynamics (MD) simulation. The simulation is performed within the XTB package[22] and the GFN-FF force field[23], enabling us to generate the various dimer configurations. The total simulation time is 50 ps, while the integration time step is 4.0 fs using a Berendsen thermostat at 298K in the NVT ensemble. We use the SHAKE algorithm to constrain bonds, for all bonds with 4 amu for the hydrogen atom mass. Then, we randomly selected 110 different configurations for the water$\cdots$water dimer and 80 for the water$\cdots$Aspirin dimers. The reference interaction energies are then calculated with DLPNO-CCSD(T)-F12/TightPNO method with the aug-cc-pvqz basis set for water$\cdots$water dimers and aug-cc-pvtz basis set for water$\cdots$Aspirin dimers.

ii. Many-body expansion of the interaction energy

The interaction energy can be decomposed into 2-body, 3-body, etc. by using the many-body expansion.[24] For example, the interaction energy of the water hexamer can be divided into K-body contributions,

$$E_{int} = E_{int}^{2-body} + E_{int}^{3-body} + \cdots + E_{int}^{6-body} \quad (5)$$

where E_{int}^{K-body} is the K-body interaction energy which can be calculated from the total energy of the subcluster of the N-mer

cluster: [24]

$$E_{int}^{K-body} = \sum_{i=1}^{K} (-1)^{K-i} \binom{N-i}{K-i} S_{tot}^{i} \quad (6)$$

where S_{tot}^{i} stands for the total energy summation of the i-th monomer subcluster.

Supplementary Note 7. Additional results for the complexes with cytosine

In Figure 4, we plot the cytosine$\cdots$water and cytosine$\cdots$cytosine interaction energy error plots, respectively. The 14 different cytosine$\cdots$cytosine configurations and reference interaction energies are from Ref. [25]. For the cytosine$\cdots$water interaction, we place two water molecules around 14 different cytosine$\cdots$cytosine structures and optimized the water molecular coordinates while fixing the cytosine$\cdots$cytosine coordinates. B3LYP functional is used for the geometry optimization. DLPNO-CCSD(T)-F12/aug-cc-pvqz with TightPNO is used as a reference cytosine$\cdots$water interaction energy with the ORCA package.[26]

Supplementary Note 8. Interactions including water

For the calculations shown in Figure 5(a), we combined energies of 145 reactions involving water. They include: the water hexamer isomerization in Figure 1(b), the water binding energy of WATER27 dataset in Figure 1(c), the water 20-mer isomerization in Figure 1(d), the water dimer stationary point geometry interaction energy in Figure 2(a), and water$\cdots$small organic molecule interaction energy in Figure 11.

	HF-r^{2}SCAN	HF-r^{2}SCAN-DC4	SC-r^{2}SCAN	SC-r^{2}SCAN-D4
MAE (all)	2.82	2.42	2.98	2.91
MoM (all)	2.77	2.32	3.24	3.10
WTMAD-2 (all)†	8.54	5.18	8.65	7.10
basic†	4.19	4.09	4.92	4.89
react.†	8.10	5.85	9.48	8.11
barriers†	7.18	8.04	13.11	13.56
inter. NCI†	13.27	4.88	10.96	6.77
intra. NCI†	11.96	4.80	8.67	5.87
		basic		
W4	6.92	6.41	3.81	3.86
G21EA	4.59	4.55	3.88	3.86
G21IP	4.56	4.54	4.66	4.64
DIPCS10	4.42	4.34	5.18	5.14
PA26	1.85	1.80	2.44	2.40
SIE4x4	12.14	12.24	17.93	17.98
ALKBDE10	4.71	4.66	5.01	5.00
YBDE18	3.89	3.67	3.89	3.36
AL2X6	1.02	1.23	0.93	1.58
HEAVYSB11	5.60	4.32	3.91	3.15
NBPRC	1.24	1.24	1.60	1.52
ALK8	1.73	1.77	2.73	2.88
RC21	2.82	2.39	4.57	4.95
G2RC	4.24	4.56	5.38	5.55
BH76RC	2.57	2.56	2.97	2.97
FH51	1.71	1.72	2.19	2.16
TAUT15	1.11	1.10	1.58	1.57
DC13	8.96	7.97	8.63	7.72
		react.		
MB16-43	10.48	10.79	12.59	14.08
DARC	3.82	2.00	3.71	2.70
RSE43	0.96	0.96	1.55	1.51
BSR36	3.20	0.16	2.32	0.48
CDIE20	1.21	1.13	1.63	1.61
ISO34	1.52	1.36	1.36	1.29
ISOL24	4.34	3.01	4.96	4.10
C60ISO	3.52	3.88	5.35	5.57
PArel	1.14	1.15	1.55	1.54
		barriers		
BH76	2.85	2.90	6.87	6.98
BHPERI	4.14	5.95	3.86	4.65
BHDIV10	3.49	3.89	5.98	6.11
INV24	1.35	1.34	1.22	1.14
BHROT27	0.62	0.64	0.76	0.76
PX13	4.74	5.03	8.75	8.83
WCPT18	2.73	3.18	5.81	5.99
		inter. NCI		
RG18	0.26	0.10	0.23	0.16
ADIM6	2.65	0.45	1.98	0.34
s22	1.55	0.42	1.18	0.24
S66	1.42	0.30	1.02	0.26
HEAVY28	0.71	0.37	0.52	0.30
WATER27	3.94	1.01	4.24	6.30
CARBHB12	0.63	0.60	0.88	1.06
PNICO23	0.71	0.29	0.64	0.76
HAL59	1.01	0.41	0.99	0.80
AHB21	0.63	0.68	1.15	1.35
CHB6	0.46	0.58	0.49	0.52
IL16	1.88	0.43	0.33	0.64
		intra. NCI		
IDISP	6.83	1.58	10.67	7.21
ICONF	0.30	0.23	0.32	0.29
ACONF	0.54	0.16	0.38	0.18
Amino20x4	0.40	0.27	0.26	0.19
PCONF	1.22	0.44	1.05	0.41
MCONF	0.94	0.21	0.63	0.45
SCONF	0.57	0.19	0.37	0.51
UPU23	1.18	0.38	0.95	0.41
BUT14DIOL	0.40	0.14	0.14	0.23

Supplementary Table 1: *Mean-absolute-error (MAE) of GMTKN55 for selected functionals for the individual datasets in the GMTKN55 database. WTMAD-2 value from Ref. [21] and mean of means (MoM) values are also noted. WTMAD-2 values for individual GMTKN55 categories †(basic properties and reaction energies for small systems (basic), reaction energies for large systems and isomerisation reactions (react.), reaction barrier heights (barriers), intermolecular noncovalent interactions (inter. NCI), and intramolecular noncovalent interactions (intra. NCI)) are also added. All units are kcal/mol.*

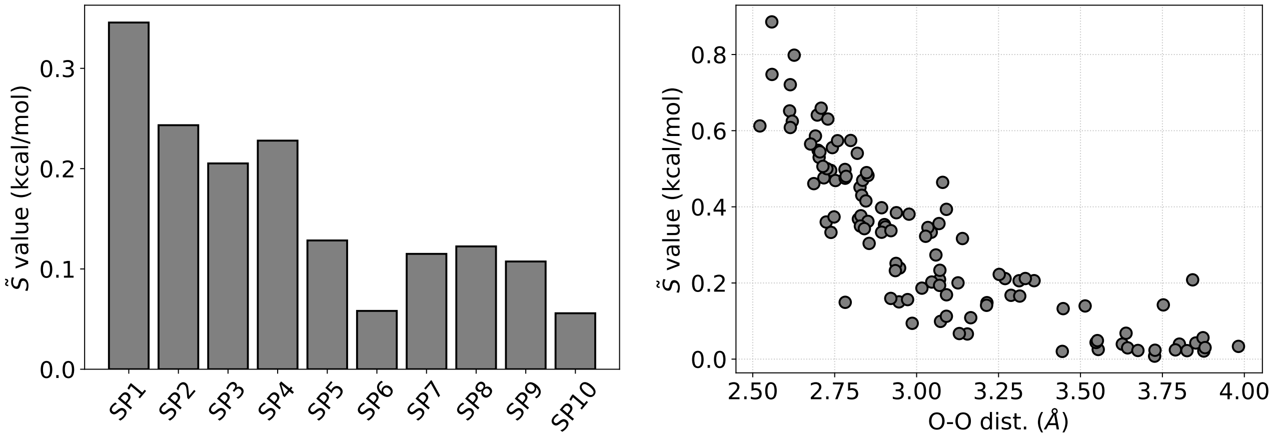

Supplementary Figure 1: *Density sensitivity plot of (left) Smith dimer configuration and (right) MD generated dimer configuration in Figure 2.*

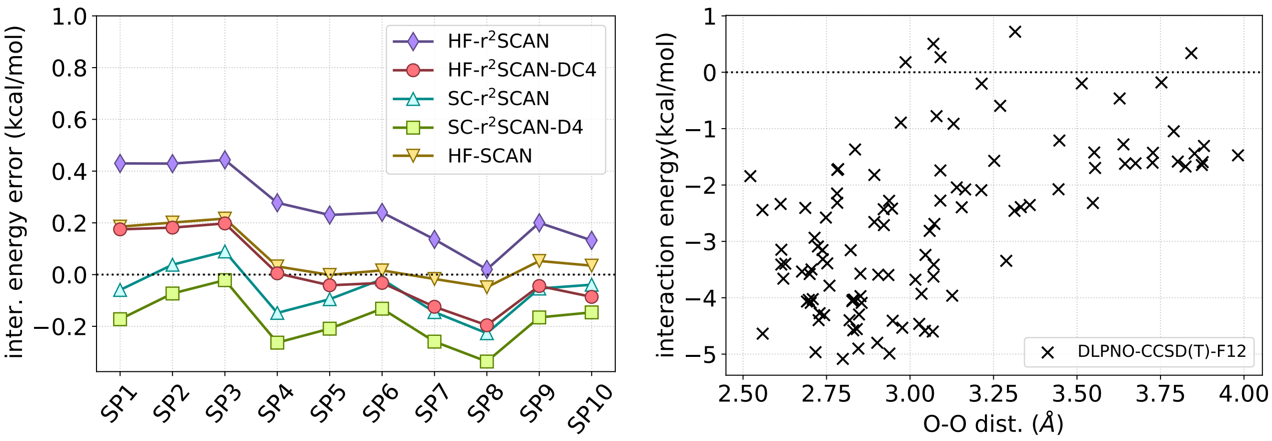

Supplementary Figure 2: *(left) Smith dimer interaction energy errors and (right) reference dimer interaction energies of MD generated dimer corresponding to Figure 2.*

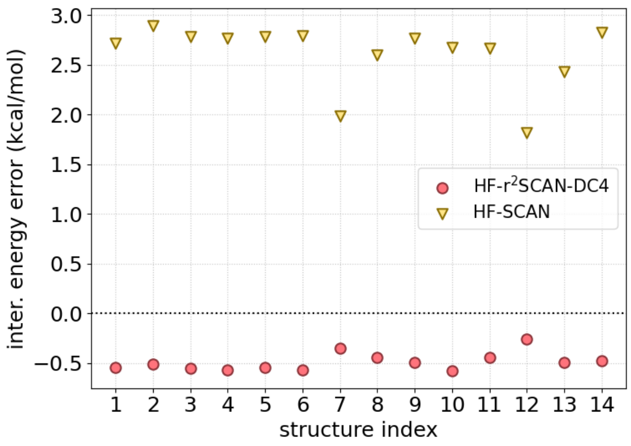

Supplementary Figure 3: *Interaction energy error plot of cytosine$\cdots$cytosine compounds for HF-r^{2}SCAN-DC4/aug-cc-pvqz and HF-SCAN. Reference HF/7Z-CP+MP2/CBS(6,7)-CP+dCC(cc-pVTZ-F12) energies are from Ref. [25].*

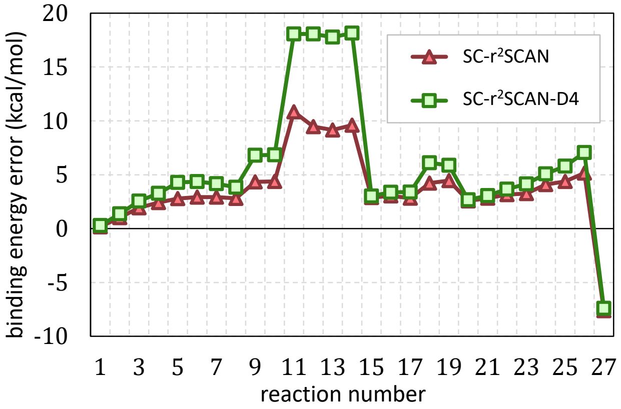

Supplementary Figure 4: *Binding energy error of WATER27 dataset for SC-r^{2}SCAN and SC-r^{2}SCAN-D4. The x-axis indicates the reaction number in WATER27 and the detailed information of reaction including geometries can be found in the GMTKN55 database.[21] D4 parameters of SC-r^{2}SCAN-D4 are from Ref. [27].*

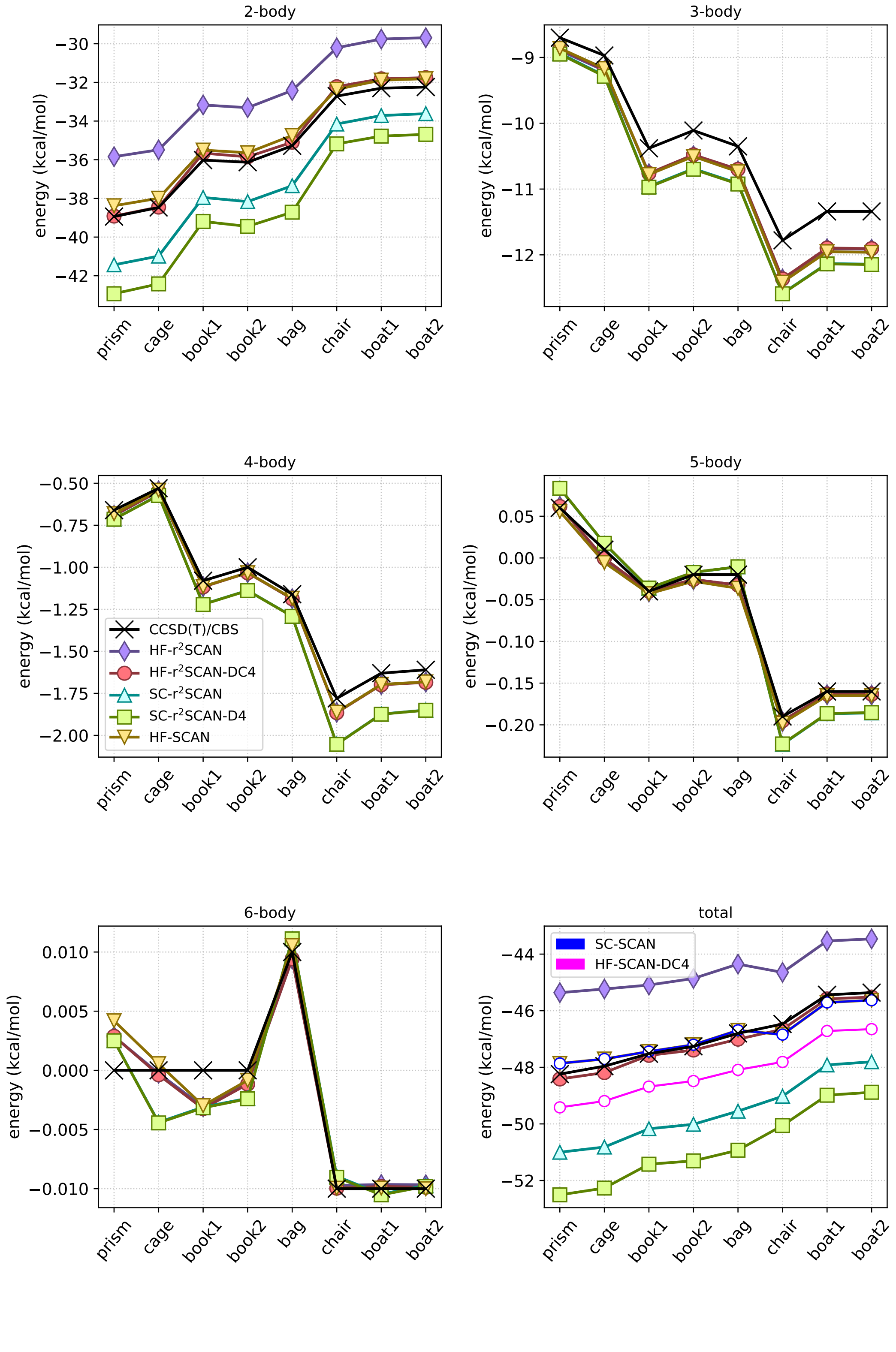

Supplementary Figure 5: *The K-body energy plot corresponding to Figure 3. For comparison, SC-SCAN and HF-SCAN-DC4 is additionally plotted in the total subplot. def2qzvppd basis set is used.*

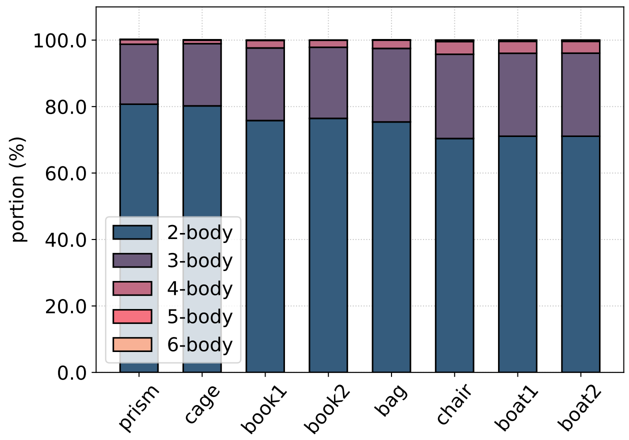

Supplementary Figure 6: *K-body percentage contributions to reference interaction energies of water hexamers (Figure 3).*

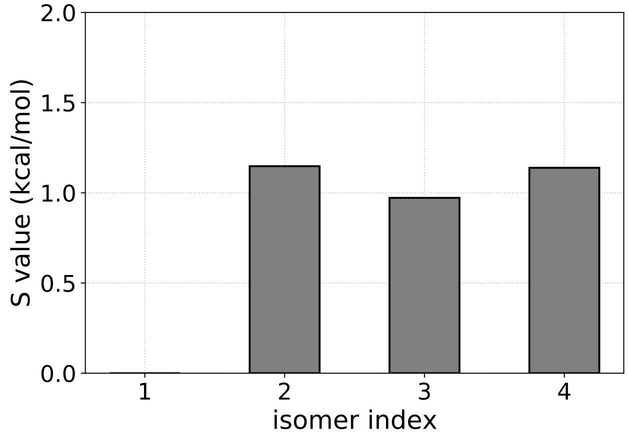

Supplementary Figure 7: *Density sensitivity $\tilde{S}$ value for isomer energies for the water 20-mers corresponding to Figure 1(d).*

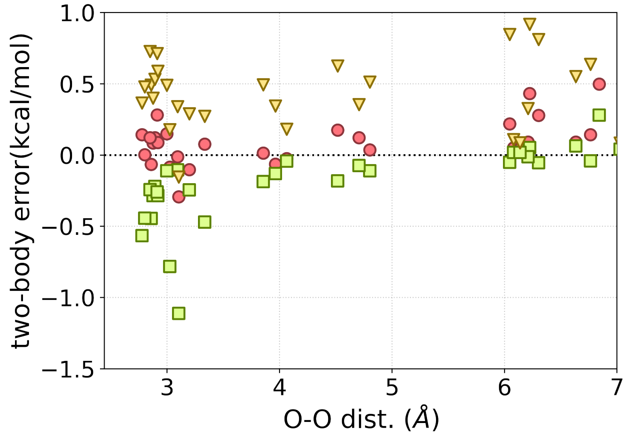

Supplementary Figure 8: *Aspirin$\cdots$water interaction including SC-r^{2}SCAN-D4. The x-axis is the oxygen$\cdots$oxygen distance between oxygen in the water and the specified oxygen in Aspirin (see inset of Fig .1(e)). aug-cc-pvtz basis set is used.*

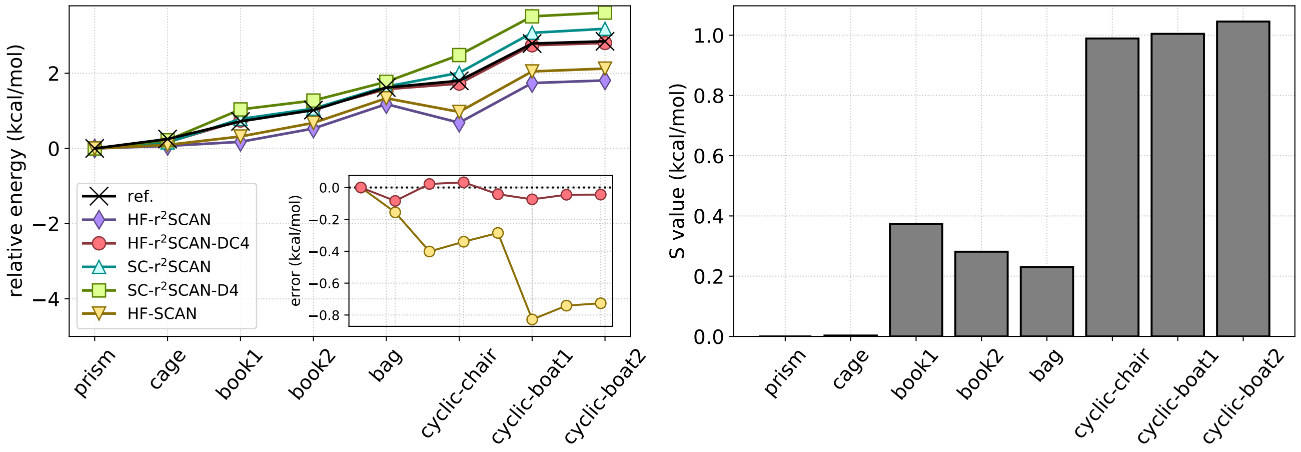

Supplementary Figure 9: *(left) Water hexamer isomer relative energy compared to its global minimum geometry prism structure; (right) their $\tilde{S}$ value calculated from the r^2SCAN functional. Geometries are from Ref. [20]. MAEs of each functional are 0.61, 0.04, 0.13, 0.36, and 0.43 kcal/mol compared to the CCSD(T)/CBS from Ref. [28]. The ordering is the same as the legend ordering. For HF-r^2SCAN-DC4, we used the dispersion parameters from Section Supplementary Note 4, whilst for SC-r^2SCAN-D4, we took the D4 parameters from Ref. [27]. The aug-cc-pvqz basis set was used for the calculations.*

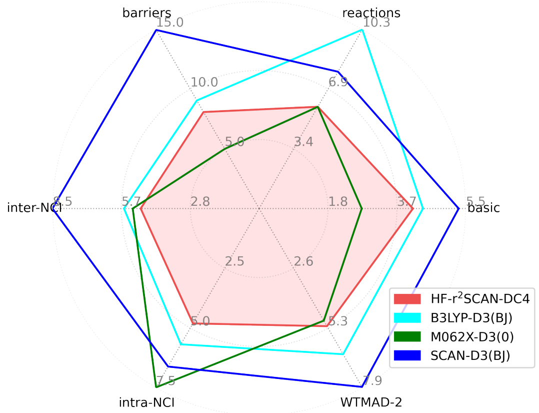

Supplementary Figure 10: *The hexagon plot with WTMAD-2 (kcal/mol) for all GMTKN55 and its categories for selected functionals. (See Ref. [21] for the detailed description of the categories).*

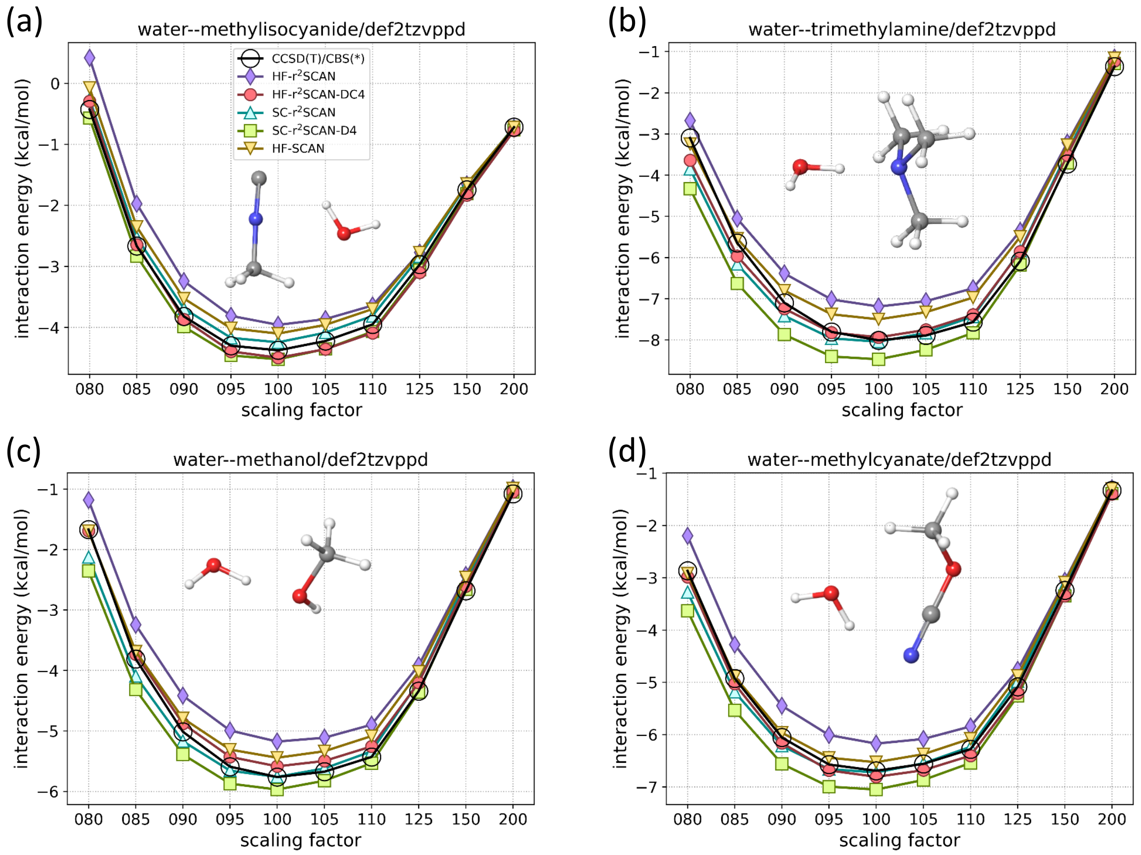

Supplementary Figure 11: *Interaction energy curve for two non-hydrogen bonded systems. Atom color code: C, gray; O, red; N, blue; and H, white. Geometries are from the HB375 dataset described in Ref. [29]. The x-axis show the scaled distance factor, f, which is used to make a translated vector t,* $t = \frac{v}{|v|}(f-1)r_{ref}$ *where v is the bond direction vector and* r_{ref} *is the distance between the hydrogen and the electron-donor atom. Detailed information about the x-axis can be found in Ref. [29].*

Supplementary References

[1] Walter Kohn and Lu Jeu Sham. Self-consistent equations including exchange and correlation effects. *Physical Review*, 140(4A):A1133, 1965.

[2] Min-Cheol Kim, Eunji Sim, and Kieron Burke. Understanding and reducing errors in density functional calculations. *Physical Review Letters*, 111(7):073003, 2013.

[3] John P Perdew, Kieron Burke, and Matthias Ernzerhof. Generalized gradient approximation made simple. *Physical Review Letters*, 77(18):3865, 1996.

[4] Stefan Vuckovic, Suhwan Song, John Kozlowski, Eunji Sim, and Kieron Burke. Density functional analysis: The theory of density-corrected dft. *Journal of Chemical Theory and Computation*, 15(12):6636–6646, 2019.

[5] Seungsoo Nam, Suhwan Song, Eunji Sim, and Kieron Burke. Measuring density-driven errors using kohn–sham inversion. *Journal of Chemical Theory and Computation*, 16(8):5014–5023, July 2020.

[6] Eunji Sim, Suhwan Song, and Kieron Burke. Quantifying density errors in dft. *Journal of Physical Chemistry Letters*, 9(22):6385–6392, 2018.

[7] Enrico Clementi and Subhas J Chakravorty. A comparative study of density functional models to estimate molecular atomization energies. *Journal of Chemical Physics*, 93(4):2591–2602, 1990.

[8] Peter MW Gill, Benny G Johnson, John A Pople, and Michael J Frisch. An investigation of the performance of a hybrid of hartree-fock and density functional theory. *International Journal of Quantum Chemistry*, 44(S26):319–331, 1992.

[9] Peter MW Gill, Benny G Johnson, John A Pople, and Michael J Frisch. The performance of the becke—lee—yang—parr (b—lyp) density functional theory with various basis sets. *Chemical Physics Letters*, 197(4-5):499–505, 1992.

[10] Gustavo E Scuseria. Comparison of coupled-cluster results with a hybrid of hartree–fock and density functional theory. *Journal of chemical physics*, 97(10):7528–7530, 1992.

[11] Nevin Oliphant and Rodney J Bartlett. A systematic comparison of molecular properties obtained using hartree–fock, a hybrid hartree–fock density-functional-theory, and coupled-cluster methods. *Journal of Chemical Physics*, 100(9):6550–6561, 1994.

[12] Suhwan Song, Stefan Vuckovic, Eunji Sim, and Kieron Burke. Density sensitivity of empirical functionals. *Journal of Physical Chemistry Letters*, 12(2):800–807, 2021.

[13] Suhwan Song, Stefan Vuckovic, Eunji Sim, and Kieron Burke. Density-corrected dft explained: Questions and answers. *Journal of Chemical Theory and Computation*, 18(2):817–827, 2022.

[14] Eunji Sim, Suhwan Song, Stefan Vuckovic, and Kieron Burke. Improving results by improving densities: Density-corrected density functional theory. *Journal of the American Chemical Society*, 144(15):6625–6639, 2022.

[15] Adam Rettig, Diptarka Hait, Luke W Bertels, and Martin Head-Gordon. Third-order møller–plesset theory made more useful? the role of density functional theory orbitals. *Journal of Chemical Theory and Computation*, 16(12):7473–7489, 2020.

[16] Jianwei Sun, Adrienn Ruzsinszky, and John P Perdew. Strongly constrained and appropriately normed semilocal density functional. *Physical Review Letters*, 115(3):036402, 2015.

[17] James W Furness, Aaron D Kaplan, Jinliang Ning, John P Perdew, and Jianwei Sun. Accurate and numerically efficient r2scan meta-generalized gradient approximation. *Journal of Physical Chemistry Letters*, 11(19):8208–8215, 2020.

[18] Eike Caldeweyher, Sebastian Ehlert, Andreas Hansen, Hagen Neugebauer, Sebastian Spicher, Christoph Bannwarth, and Stefan Grimme. A generally applicable atomic-charge dependent london dispersion correction. *Journal of Chemical Physics*, 150(15):154122, 2019.

[19] Stefan Grimme, Jens Antony, Stephan Ehrlich, and Helge Krieg. A consistent and accurate ab initio parametrization of density functional dispersion correction (dft-d) for the 94 elements h-pu. *Journal of Chemical Physics*, 132(15):154104, 2010.

[20] Sandeep K Reddy, Shelby C Straight, Pushp Bajaj, C. Huy Pham, Marc Riera, Daniel R Moberg, Miguel A Morales, Chris Knight, Andreas W Götz, and Francesco Paesani. On the accuracy of the mb-pol many-body potential for water: Interaction energies, vibrational frequencies, and classical thermodynamic and dynamical properties from clusters to liquid water and ice. *Journal of Chemical Physics*, 145(19):194504, 2016.

[21] Lars Goerigk, Andreas Hansen, Christoph Bauer, Stephan Ehrlich, Asim Najibi, and Stefan Grimme. A look at the density functional theory zoo with the advanced gmtkn55 database for general main group thermochemistry, kinetics and noncovalent interactions. *Physical Chemistry Chemical Physics*, 19(48):32184–32215, 2017.

[22] Christoph Bannwarth, Sebastian Ehlert, and Stefan Grimme. Gfn2-xtb—an accurate and broadly parametrized self-consistent tight-binding quantum chemical method with multipole electrostatics and density-dependent dispersion contributions. *Journal of Chemical Theory and Computation*, 15(3):1652–1671, 2019.

[23] Sebastian Spicher and Stefan Grimme. Robust atomistic modeling of materials, organometallic, and biochemical systems. *Angewandte Chemie International Edition*, 59(36):15665–15673, 2020.

[24] Urszula Góra, Rafał Podeszwa, Wojciech Cencek, and Krzysztof Szalewicz. Interaction energies of large clusters from many-body expansion. *Journal of Chemical Physics*, 135(22):224102, 2011.

[25] Holger Kruse and Jiri Sponer. Revisiting the potential energy surface of the stacked cytosine dimer: Fno-ccsd (t) interaction energies, sapt decompositions, and benchmarking. *Journal of Physical Chemistry A*, 123(42):9209–9222, 2019.

[26] Frank Neese, Frank Wennmohs, Ute Becker, and Christoph Riplinger. The orca quantum chemistry program package. *Journal of Chemical Physics*, 152(22):224108, 2020.

[27] Sebastian Ehlert, Uwe Huniar, Jinliang Ning, James W Furness, Jianwei Sun, Aaron D Kaplan, John P Perdew, and Jan Gerit Brandenburg. r2scan-d4: Dispersion corrected meta-generalized gradient approximation for general chemical applications. *Journal of Chemical Physics*, 154(6):061101, 2021.

[28] A Otero-De-La-Roza and Erin R Johnson. Non-covalent interactions and thermochemistry using xdm-corrected hybrid and range-separated hybrid density functionals. *Journal of Chemical Physics*, 138(20):204109, 2013.

[29] Jan Rezác. Non-covalent interactions atlas benchmark data sets: Hydrogen bonding. *Journal of Chemical Theory and Computation*, 16(4):2355–2368, 2020.
