# Supplementary material for: Extending density functional theory with near chemical accuracy beyond pure water
Source: Nat Commun. 2023 Feb 13;14:799. doi: 10.1038/s41467-023-36094-y (PMC9925738; doi:10.1038/s41467-023-36094-y)
Supplement: Supplementary file 4 — Description of Additional Supplementary files [file 41467_2023_36094_MOESM4_ESM.rtf]

Data1: Individual reaction energy and density sensitivity values of HF-r2SCAN for GMTKN55 reported in this study.
Data2: DLPNO-CCSD(T)-F12/TightPNO interaction energies and cartesian coordinates of cytosine¡¤ ¡¤ ¡¤ water, aspirin¡¤ ¡¤ ¡¤ water, and water¡¤ ¡¤ ¡¤ water dimers.
